# Effects of exercises based on ACSM recommendations on patients with heart failure with preserved ejection fraction: a systematic review and meta-analysis of randomized controlled trials

**DOI:** 10.3389/fphys.2026.1838821

**Published:** 2026-07-09

**Authors:** Xiaojing Shi, Fang Liu, Ruirui Song, Xuefeng Guo, Jian Huang, Jun Chen, Hongmei Gao

**Affiliations:** Cardiology, The Second Affiliated Hospital of Shandong University of Traditional Chinese Medicine, Jinan, China

**Keywords:** American College of Sports Medicine, cardiac function, exercise training, heart failure with preserved ejection fraction, meta-analysis

## Abstract

**Objective:**

This study aimed to evaluate the effects of exercise training following the American College of Sports Medicine (ACSM) guidelines on exercise capacity, quality of life, and left ventricular function in patients with heart failure with preserved ejection fraction (HFpEF).

**Methods:**

PubMed, Embase, Web of Science, and the Cochrane Library were searched for randomized controlled trials (RCTs) comparing exercise interventions with usual care in patients with HFpEF. Primary outcomes included exercise capacity (6-MWT, Peak VO_2_, quality of life (total score of MLHFQ), LVEF, and diastolic function indicator (E/e’ ratio). The included studies were subgrouped for analysis into a high-adherence group (≥70%) and a low/uncertain-adherence group based on their compliance with the ACSM exercise recommendations.

**Results:**

A total of 19 RCT involving 1490 participants were included in this meta-analysis. Pooled results demonstrated that exercise training compliant with ACSM guidelines significantly improved exercise capacity, including the 6-MWT (SMD = 0.82, 95% CI: 0.34 to 1.30, P = 0.0008) and Peak VO_2_ (SMD = 0.63, 95% CI: 0.36 to 0.91, P < 0.00001). The intervention also markedly enhanced patients’ quality of life reflected by the MLHFQ total score (SMD = −0.52, 95% CI: −0.84 to −0.20, P = 0.05). Marginally significant improvements were observed in left ventricular ejection fraction (SMD = 0.19, 95% CI: 0.00 to 0.38, P = 0.05) and E/e’ ratio (SMD = −0.14, 95% CI: −0.29 to 0.00, P = 0.05).Subgroup analyses stratified by adherence to ACSM recommendations (≥70% vs. <70%) showed that the high-adherence group achieved statistically greater improvements in 6-MWT (P = 0.03) and peak VO_2_ (P = 0.003). Although the high-adherence group obtained larger effect sizes for MLHFQ scores and LVEF, the between-group differences did not reach statistical significance. No significant intergroup differences were found in other diastolic function indicators.

**Conclusion:**

This study indicates that implementing the ACSM-recommended exercise regimen with high adherence can effectively improve exercise capacity, quality of life, and certain left ventricular function parameters in patients with HFpEF. Stratified analysis by ACSM adherence enriches evidence for individualized HFpEF exercise rehabilitation and guides clinical exercise strategy optimization.

## 1 Introduction

Heart failure represents the severe manifestation or end-stage of various cardiac diseases. The prevalence of heart failure among adults in developed countries ranges from 1.0% to 2.0% (2018; Virani et al., 2020), and it is continuously increasing (Dunlay and Roger, 2014; Golla et al., 2024). Moreover, mortality and rehospitalization rates remain high (Cheng et al., 2014), and heart failure leads to a decline in patients’ quality of life (Lewis et al., 2007). Consequently, heart failure is recognized as a growing public health burden (Cook et al., 2014).Heart failure with preserved ejection fraction (HFpEF) accounts for more than 50% of heart failure cases in patients aged 65 and older (Gerber et al., 2015; Pfeffer et al., 2019). The morbidity and mortality rates of HFpEF are comparable to those of heart failure with reduced ejection fraction (HFrEF) (Edelmann et al., 2011a; Edelmann et al., 2011b; Lauritsen et al., 2018). A hallmark feature of HFpEF is exercise intolerance, characterized by severe exertional dyspnea and fatigue (Upadhya et al., 2015; Tucker et al., 2020).In contrast to the numerous guideline-recommended pharmacological and non-pharmacological treatment options available for HFrEF, effective therapies for improving clinical outcomes in HFpEF have been limited. To date, only the sodium-glucose cotransporter 2 inhibitor empagliflozin (Sakai and Miura, 2019), the glucagon-like peptide-1 receptor agonist semaglutide (Kosiborod et al., 2024), and the non-steroidal mineralocorticoid receptor antagonist finerenone (Yang et al., 2025)have demonstrated improvements in clinical outcomes for HFpEF patients. Within this context, non-pharmacological interventions have become a key direction for improving the functional status of HFpEF patients. Among these, exercise intervention has garnered widespread attention due to its low cost, high safety profile, and multifaceted benefits.

Exercise training (ET) has been shown to improve physical performance metrics in HFpEF patients, including peak oxygen uptake (VO_2_ peak) and six-minute walk test (6MWT) distance (Gary et al., 2004; Kitzman et al., 2010; Smart et al., 2012; Kitzman et al., 2013, Kitzman et al., 2016; Donelli Da Silveira et al., 2020). In recent years, numerous studies have begun to investigate the effects of different exercise doses and durations on cardiopulmonary reserve function, exercise capacity, and quality of life in HFpEF patients. However, the findings have been inconsistent. For instance, a study by Donelli Da Silveira et al. showed that both high-intensity interval training and moderate-intensity continuous training groups improved patients’ peak VO_2_ and quality of life scores (Donelli Da Silveira et al., 2020). In contrast, a study by Mueller et al. demonstrated no statistically significant difference in the change of peak VO_2_ between high-intensity interval training and moderate-intensity continuous training groups (Mueller et al., 2021). Therefore, the impact of exercise intensity on HFpEF patients warrants further investigation.

The American College of Sports Medicine (ACSM), a leading global authority in sports medicine, has issued Guidelines for Exercise Prescription, which provide standardized exercise prescriptions for patients with cardiovascular diseases. These prescriptions include recommendations for aerobic exercise, resistance exercise, and flexibility exercise, detailing the frequency, intensity, time, and type for each exercise modality (Garber et al., 2011).

Consequently, we conducted a systematic review of existing studies to investigate whether exercise therapy adhering to ACSM recommendations yields superior outcomes compared to interventions with low or uncertain adherence. The findings of this study aim to address the current evidence gap in the field of exercise intervention for HFpEF, provide an evidence-based foundation for the clinical application of ACSM exercise recommendations in HFpEF patients, and offer data support for developing individualized exercise rehabilitation guidelines for HFpEF patients.

## 2 Materials and methods

Data sources, search strategies, data acquisition, inclusion and exclusion criteria, outcome measures, quality assessment, and statistical methods in this report were conducted following the Preferred Reporting Items for Systematic Reviews and Meta-analyses (PRISMA) guidelines (Page et al., 2021). The protocol for this systematic review and meta-analysis has been registered on the International Platform of Registered Systematic Review and Meta-analysis Protocols (INPLASY: 202630070). The data supporting this article can be found in the article and its online **Supplementary Material**.

### 2.1 Search strategy

Four databases (PubMed, EMBASE, Web of Science, and Cochrane Library) were searched from their inception to November 2025. The search strategy was constructed around the PICOS tool (P) population: patients with preserved ejection fraction; (I) intervention: exercises; (C) control group: no exercise or usual care group; (O) outcomes: 6-minute walk test(6-MWT), peak oxygen consumption (peakVO_2)_, total score of the Minnesota Living with Heart Failure Questionnaire (MLWHQ), left ventricular ejection fraction (LVEF), the ratio of mitral early diastolic inflow velocity to mitral annular early diastolic velocity (E/e’ ratio), early diastolic transmitral flow velocity/Atrial systolic transmitral flow velocity ratio (E/A ratio), ventilation/Carbon Dioxide production slope (VE/VCO_2_ slope) and Deceleration time (DT); (S) study type: randomized controlled trial (RCT). References and related literature listed in the identified studies were also evaluated to broaden the search. The detailed retrieval strategy is shown in **Table 1** (using PubMed as an example).

**Table 1:** Search strategy on PubMed.

Search	Query
#1	"Heart Failure, Diastolic"[Mesh]
#2	"heart failure diastolic"[Title/Abstract] OR "diastolic heart failures"[Title/Abstract] OR "diastolic heart failure"[Title/Abstract] OR "heart failure preserved ejection fraction"[Title/Abstract] OR "heart failure normal ejection fraction"[Title/Abstract]
#3	#1 OR #2
#4	"Exercise"[MeSH Terms]
#5	"Exercise"[Title/Abstract] OR "Walking"[Title/Abstract] OR "nordic walking"[Title/Abstract] OR "Exercises"[Title/Abstract] OR "physical activity"[Title/Abstract] OR "activities physical"[Title/Abstract] OR "activity physical"[Title/Abstract] OR "physical activities"[Title/Abstract] OR "exercise physical"[Title/Abstract] OR "exercises physical"[Title/Abstract] OR "physical exercise"[Title/Abstract] OR "physical exercises"[Title/Abstract] OR "exercise aerobic"[Title/Abstract] OR "aerobic exercise"[Title/Abstract] OR "aerobic exercises"[Title/Abstract] OR "exercises aerobic"[Title/Abstract] OR "exercise training"[Title/Abstract] OR "exercise trainings"[Title/Abstract] OR "training exercise"[Title/Abstract] OR (("education"[MeSH Subheading] OR "education"[All Fields] OR "Training"[All Fields] OR "education"[MeSH Terms] OR "train"[All Fields] OR "train s"[All Fields] OR "trained"[All Fields] OR "training s"[All Fields] OR "Trainings"[All Fields] OR "trains"[All Fields]) AND "Exercise"[Title/Abstract]) OR "training resistance"[Title/Abstract] OR "strength training"[Title/Abstract] OR "training strength"[Title/Abstract] OR "Balance"[Title/Abstract] OR "Ambulation"[Title/Abstract] OR "stair climbing"[Title/Abstract] OR "walking nordic"[Title/Abstract] OR "pole walking"[Title/Abstract] OR "walking pole"[Title/Abstract]
#6	#5 OR #4
#7	#6 AND #3

### 2.2 Inclusion criteria and exclusion criteria

#### 2.2.1 Inclusion criteria

The following criteria were used for inclusion: (a) Published RCTs; (b) Study participants were adult patients with HFpEF, with LVEF ≥ 45%; (c) The intervention for the experimental group was exercise, including aerobic or endurance exercise, resistance training, high-intensity interval training (HIIT), flexibility training, or a combination of the above; (d) The control group received no treatment or received treatment unrelated to exercise; (e) All participants did not receive specific pharmacotherapy; (f) The outcome measures in the study included one or more of the following: 6-MWT, peakVO_2_, total score of MLWHF, LVEF, E/e’ ratio, E/A ratio, VE/VCO_2_ slope and DT.

#### 2.2.2 Exclusion criteria

Studies were excluded if they met the following criteria: (a) Studies published as reviews or conference abstracts; (b) The control group received exercise as an intervention; (c) Study participants took specific medications during the exercise intervention; (d) Intervention duration of less than 4 weeks; (e) Patients had acute heart failure; (f) Patients were minors; (g) Patients had heart failure with reduced ejection fraction (HFrEF).

### 2.3 Data screening and extraction

Two authors (J.S. and R.S.) independently performed the literature screening and data extraction. After initial title/abstract screening, potentially eligible studies underwent full-text review. Any disagreements were resolved by consensus with a third author (H.G.). There were no restrictions on age, gender, publication date, or language. Data on study characteristics, participants, exercise interventions, and outcomes were extracted into a predefined spreadsheet.

Subsequently, two other authors (X.G. and J.C.) independently evaluated the exercise intervention dose in each study for adherence to ACSM recommendations for HFpEF (**Table 2**). Each exercise parameter (frequency, intensity, etc.) was scored (2=meets standard, 1=uncertain, 0=does not meet). Discrepancies were resolved by a third author (F.L.). The overall adherence proportion was calculated. Studies with ≥70% adherence were classified as “high adherence”; those with <70% were “low/uncertain adherence.”

**Table 2:** ACSM exercise recommendations for heart failure patients.

Exercise dose	Cardiorespiratory exercise	Resistance exercise	Flexibility exercise
Frequency	3–5 days per week	2–3 days per week	2–3 days per week
Intensity/workload	40-59%HRR; 57-76%HRmax; 37-63%peakVO2 RPE of 12-14 on a 6–20 scale	40%–50% 1RM RPE of 11-13 on a 6–20 scale	Stretch until you feel your muscles being pulled tight or a slight discomfort
Duration	Continuous or cumulative 30 min	1-2 group, 8-12repetitions	Keep static pulling for 10–30 s; repeat 2–4 time

HRR, Reserve heart rate; peakVO_2_, peak oxygen uptake; HRmax, Maximum heart rate; RPE, Rating of perceived exertion; 1RM, Maximum power of 1 repetition.

### 2.4 Risk of bias of individual studies

Two researchers (J.H. and G.X.) assessed the risk of bias independently. The bias of the studies was evaluated using Cochrane Handbook version 5.1.0.The following seven aspects were considered: (1) random sequence generation (selection bias), (2) allocation concealment (selection bias), (3) blinding of participants and personnel (performance bias), (4) blinding of outcome assessment (detection bias), (5) incomplete outcome data (attrition bias), (6) selective reporting (reporting bias), and (7) other sources of bias. Trials were categorized into three levels of risk of bias by the number of components for which high risk of bias potentially existed: high risk (five or more), moderate risk (three or four), and low risk (two or less) (Higgins et al., 2011).

### 2.5 Data analysis

RevMan 5.4 software was used for meta-analysis. The effect of the exercise recommended by ACSM on patients with HFpEF was evaluated by standardized mean difference (SMD) and 95% confidence interval (CI).Between-study heterogeneity was assessed via the I^2^ statistic, following Higgins et al. (2003): low (0%–25%), moderate (25%–50%), substantial (50%–75%), and considerable heterogeneity (75%–100%) (Higgins et al., 2003). We used a random-effects (RE) model for I^2^ ≥ 50% and a fixed-effects (FE) model for I^2^ < 50%. The 95% prediction interval (PI) was additionally calculated using Stata18.0 software based on the method proposed by Higgins et al. to evaluate the variation of treatment effects across future individual studies. Heterogeneity across studies was further investigated by performing sensitivity analyses. A p-value<0.05 was considered to be associated with the occurrence of the outcome.

## 3 Results

### 3.1 Study identification and selection

**Figure 1:**
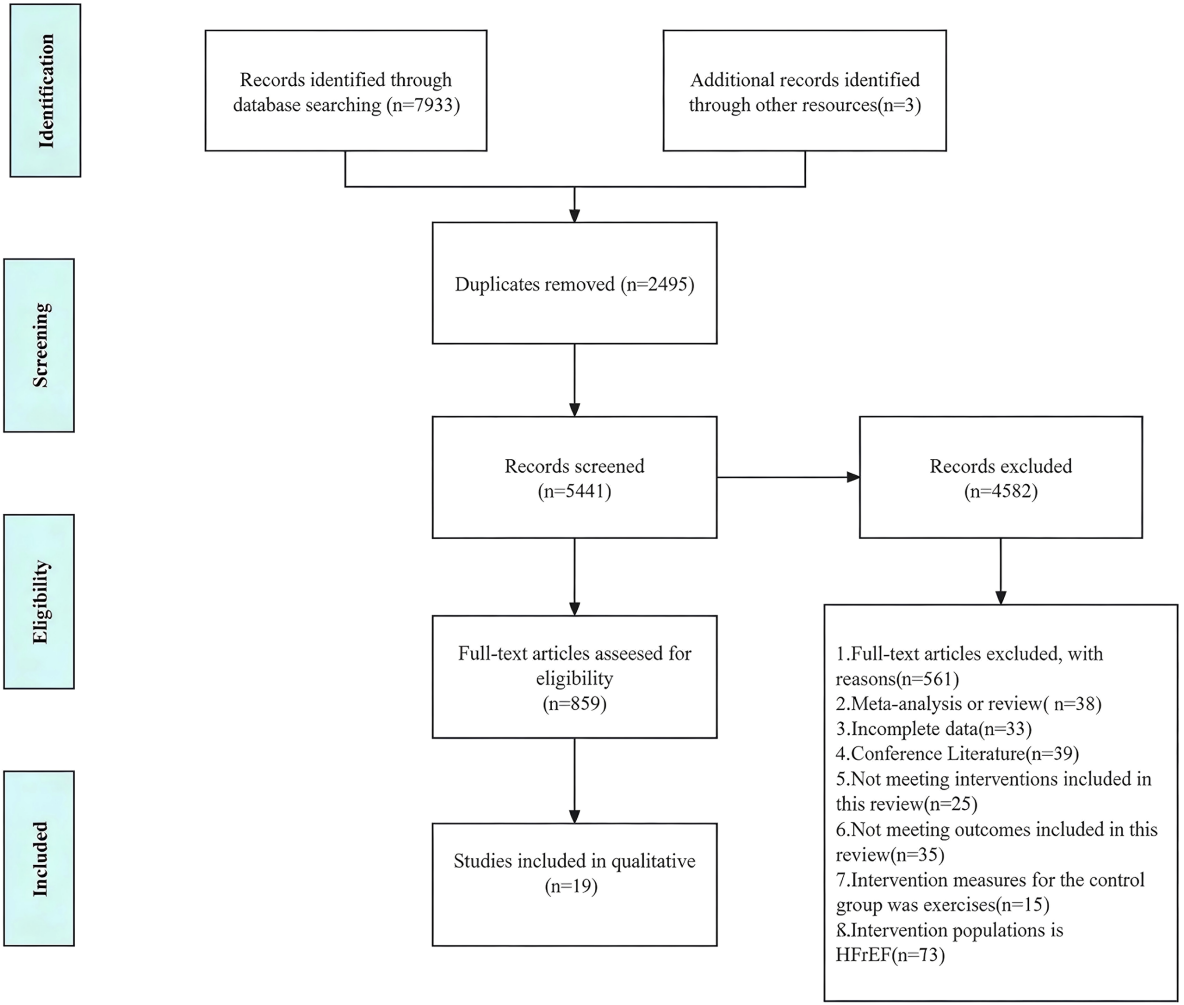
Flow diagram of literature selection.

A total of 7933 articles were retrieved from electronic databases, with an additional 3 articles obtained through manual retrieval. After removing duplicate records, the titles and abstracts of the remaining 5441 articles were screened, and 4582 articles were excluded once more. The remaining 859 articles were then fully reviewed (in full text), and another 839 articles were excluded for the following reasons: being reviews or systematic reviews, having incomplete data, being conference papers, involving intervention measures unrelated to exercise, being non-randomized controlled trials (non-RCTs), focusing on patients with heart failure with reduced ejection fraction (HFrEF), having control groups that received exercise interventions, and lacking the required outcome measures, etc. This final screening process resulted in 19 articles being included in this study (**Figure 1**).

### 3.2 Quality assessment of the included studies

These 19 articles included 20 comparative studies, with a total of 1490 participants—754 in the experimental group and 736 in the control group (Gary et al., 2004; Gary and Lee, 2007; Andryukhin et al., 2010; Kitzman et al., 2010; Edelmann et al., 2011a; Alves et al., 2012; Haykowsky et al., 2012; Smart et al., 2012; Kitzman et al., 2013; Nolte et al., 2015; Fu et al., 2016; Maldonado-Martín et al., 2017; Lang et al., 2018; Brubaker et al., 2020; Hassanein et al., 2021; Mueller et al., 2021; Alonso et al., 2022; Li et al., 2024; Edelmann et al., 2025). One of the studies involved two exercise intervention groups. These studies were conducted in multiple countries, including Spain, the United States, Germany, Portugal, China, Egypt, Australia, Japan, the UK, and Russia (**Table 3**).

**Table 3:** Overview of general characteristics of the study and participants.

Author	Country	Year	Population	Age mean (SD)	Total/male/female	Total/C/T	Intervention	Duration weeks	Control	Outcome
Sara	Spain	2020	HFpEF LVEF≥50%	NR	47/6/41	47/24/23	cycling and walking	16	CON	6-MWT peakVO_2_
Gary	US	2004	LVEF≥45%	T:67(11) C:69(11)	32/0/32	32/16/16	Walking	12	CON	6-MWT Total score of MLHFQ
Gary	US	2007	HFpEF, LVEF 56 ± 7%	68(12)	23/0/23	23/10/13	Walking	12	CON	6-MWT Total score of MLHFQ
Alves	Portugal	2012	HFpEF, LVEF≥55%	62.9(10.2)	31/22/9	31/11/20	aerobic exercise: on treadmill or bicycle ergometer	24	CON	LVEF E/A radio DT
Tieh-Cheng	China	2015	HFpEF, LVEF≥50%	T:63.1(2.6) C:60.5(2.7)	T:30/18/12 C:30/20/10	60/30/30	bicycle ergometer	12	CON	LVEF E/e’ radio E/A radio Total score of MLHFQ VE/VCO_2_ slope
Mueller	Germany	2021	HFpEF, LVEF≥50%	HIIT:70(7) MCT:70(8) C:69(10)	HIIT:58/17/41 MCT:58/23/35 C:60/19/41	HIIT:118/58/60 MCT:118/58/60	1.High-intensity interval training 2.moderate continuous training	52	CON	peakVO_2_ VE/VCO_2_ slope E/e’ radio LAVI
Hassanein	Egypt	2021	HFpEF, LVEF≥50%	T:57.47(6.1) C:58.5(6.31)	T:30/11/19 C:30/13/17	60/30/30	treadmill	12	CON	E/A radio E/e’ radio LVEF 6-MWT Total score of MLHFQ
Kitzman	US	2010	HFpEF, LVEF≥50%	T:70(6) C:69(5)	T:26/6/20 C:27/7/20	53/27/26	walking on a track/cycling on a Schwinn Airdyne	16	CON	VE/VCO_2_ slope 6-MWT Total score of MLHFQ
Kitzman	US	2013	HFpEF, LVEF≥50%	T:70(7) C:70(7)	T:32/9/23 C:31/6/25	63/31/32	walking on a track/cycling on a Schwinn Airdyne	16	CON	PeakVO_2_ VE/VCO_2_ slope 6-MWT
Smart	Australia	2012	HFpEF, LVEF>45%	T:67(5.8) C:61.9(6.9)	T:12/7/5 C:13/6/7	25/13/12	bicycle ergometer	16	CON	Peak VO_2_ VE/VCO_2_ slope
Peter	US	2021	HFpEF, LVEF≥50%	T:70.3(6.7) C:69.2(6.2)	T:58/14/44 C:58/8/50	116/58/58	walking on an indoor track/Schwinn Airdyne cycle ergometry	16	CON	PeakVO2 6-MWT
Nolte	Germany	2014	HFpEF, LVEF≥50%	T:64(8) C:65(6)	T:44/20/24 C:20/8/12	64/20/44	aerobic endurance training (cycling) Resistance training	12	CON	E/A radio DT VE/VCO_2_ slope
Edelmann	Germany	2012	HFpEF, LVEF≥50%	T:64(8) C:65(6)	T:44/20/24 C:20/8/12	64/20/44	aerobic endurance training (cycling) Resistance training	12	CON	LVEF 6-MWT Total score of MLHFQ
Edelman	Germany	2025	HFpEF, LVEF≥50%	T:69.1 (7.4) C:70.1 (7.1)	T:161/61/100 C:161/69/92	322/161/161	bicycle ergometer resistance training	52	CON	peakVO_2_ E/e’ radio
Haykowsky	Canada	2012	HFpEF, LVEF≥50%	T:70(6) C:68(5)	T:22/4/18 C:18/17/1	40/18/22	walking on a track/cycling on a Schwinn Airdyne	12	CON	peakVO_2_
Jingen	China	2024	HFpEF, LVEF≥50%	T:60.33 (8.78) C:60.8(9.7)	T:60/50/10 C:60/47/13	120/60/60	Baduanjin	12	CON	peakVO_2_ 6-MWT LVEF Total score of MLHFQ
Chim	UK	2018	HFpEF, LVEF≥45%	T:71.8(9.9) C:76(6.6)	T:25/9/16 C:25/14/11	50/25/25	chair-based exercise Progressive walking	24	CON	Total score of MLHFQ
Andryukhin	Russia	2010	HFpEF, LVEF>50%	T:68 (8.15) C:68 (11.11)	T:44/12/32 C:41/14/27	85/41/44	muscles sitting or standing positions/walking	52	CON	Total score of MLHFQ 6-MWT
Windy	Russia	2022	HFpEF, LVEF≥50%	T:63.3(9.4) C:65.6(9.3)	T:25/14/11 C:34/18/16	59/25/34	aerobic exercise/resistance training	72	CON	6-MWT

The interventions in the experimental group included various exercise modalities, such as cycling, walking, resistance training, aerobic exercise, high-intensity interval training (HIIT), treadmill exercise, Baduanjin (a traditional Chinese Qigong exercise), and chair-based exercise. The duration of the interventions ranged from 12 weeks to 72 weeks. Participants in the control group did not receive any exercise intervention. According to the standards of the American College of Sports Medicine (ACSM), among the 20 comparative studies, 20addressed aerobic exercise dose, 5 addressed resistance exercise dose, and 3 addressed flexibility exercise dose. Fourteen demonstrated an adherence rate exceeding 70% to the American College of Sports Medicine (ACSM) recommendations, while six studies had an adherence rate below 70% (see **Table 4**).

**Table 4:** Assessment of ACSM adherence.

Author year	Aerobic exercise						Resistance exercise								Flexibility exercise						ACSM adherence	
	Frequency/ adherence		Intensity/ adherence		Duration/ adherence		Frequency/ adherence		Intensity/ adherence		Repetitions/ adherence		Sets/ adherence		Frequency/ adherence		Intensity/ adherence		Duration/ adherence		Points	Percent
Sara 2020	3d/w	J	50-70%peak VO2	K	60 min	J									3times/d	J	NR	K	10min	K	9/12	75%
Gary 2004	3d/w	J	40-60%THR	K	30min	J															5/6	83%
Gary 2007	3d/w	J	40-60%THR	K	30min	J															5/6	83%
Alves 2012	3 times/w	J	70%–75%HRmax	J	35-55min	J									3times/d	J	NR	K	10min	K	10/12	83%
Tieh-Cheng2015	3 times/w	J	30-80%HRR 30-80%peakVO2	K	33min	J															5/6	83%
Mueller (HIIT) 2021	3times/w	J	HIIT: 80-90%HRR, RPE 15-17	L	38min	J															4/6	67%
Mueller (MCT) 2021	5times/w	J	MCT: 35-50%HRR, RPE 11-13	J	40min	J															6/6	100%
Kitzman 2013	3times/w	J	40-50%HRR, gradually increase to 70%HRR	K	30-60min	J															5/6	83%
Kitzman 2010	3times/w	J	40-70%HRR	K	30-60min	J															5/6	83%
Smart2012	3times/w	J	60-70%peak VO2	L	30min	J															4/6	67%
Peter2021	3times/w	J	40-60%HRR	K	60min	J									3times/d	J	NR	K	10min	K	9/12	75%
Edelmann 2025	2-3times/w	K	50-70%peak VO2	K	30-60min	J	2-3times/w	J	60% 1RM	L	12-15reps	K	1-2Sets	J							9/14	64%
Edelmann 2012	2-3times/w	K	50-70%peak VO2	K	30-60min	J	2-3times/w	J	60% -65%1RM	L	12-15reps	K	1-2Sets	J							9/14	64%
Nolte2014	2-3times/w	K	50-70%peak VO2	K	20-40min	K	2times/w	J	60%- 65% 1RM	L	15reps	L	1Sets	J							7/14	50%
Jingen2024	3times/w	J	NR	K	45min	J															5/6	83%
Haykowsky 2012	3times/w	J	40%HRR, gradually increase to 70%HRR	K	60min	J															5/6	83%
Chim2018	2-3times/w	K	70% of the mean MET score	K	13-40min	K	7times/w	L	NR	K	10-15reps	K	2SETS	J							7/14	50%
Andryukhin 2010	4ties/w	J	NR	K	30min	J															5/6	83%
Windy2022	2-3imes	K	40-80%HRR; More than 120min/week	K	30min	J	2-3times	J	NR	K	10-15reps	K	1SETS	J							10/14	71%
Hassanein 2021	3times/w	J	40-75%HRR	K	60min	J															5/6	83%

## 4 Methodological quality assessment

**Figure 2:**
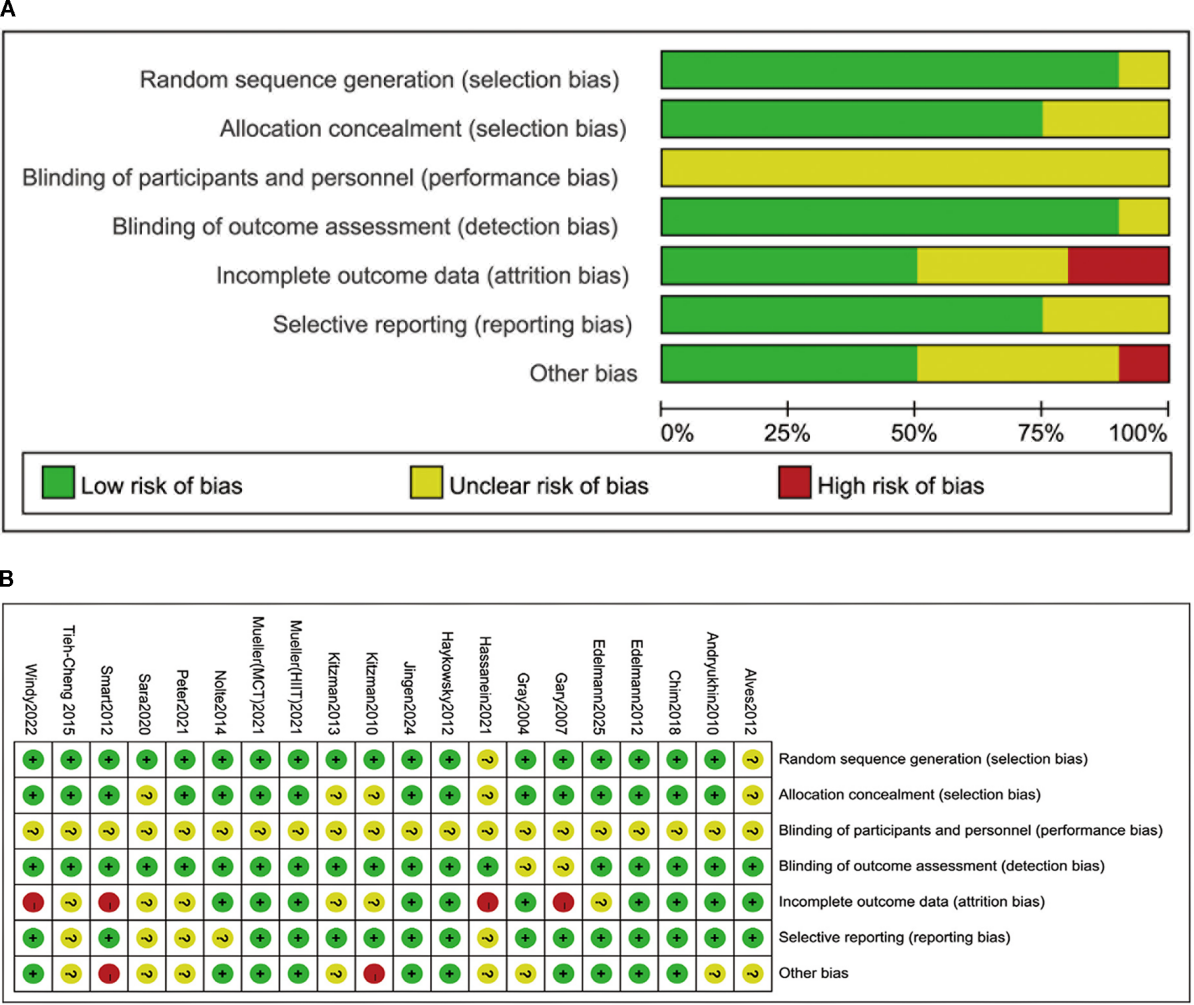
**(A)** Risk of bias graph based on the Cochrane risk-of-bias tool. Seven bias domains were assessed: random sequence generation, allocation concealment, blinding of participants and personnel, blinding of outcome assessment, incomplete outcome data, selective reporting, and other bias. Bars represent the percentage of included trials judged as low risk, unclear risk, or high risk of bias for each domain. **(B)** Risk of bias summary graph based on the Cochrane risk-of-bias tool. Horizontally, it lists 7 criteria for bias assessment: random sequence generation, group concealment, blinding of subjects and researchers, blinding of outcome assessment, incomplete outcome data, selective reporting, and other biases; vertically, it shows 19 included studies (divided into 20 groups), presenting intuitively the individual risk assessment results of each study in each bias dimension.

A risk of bias assessment was conducted for all included studies. All 19 studies were randomized controlled trials. Seventeen studies reported the method of random sequence generation, and 14 articles described their allocation concealment. Due to the inherent difficulty in implementing double-blinding for exercise interventions, 4 articles reported using single-blind methods, while the blinding status was unclear in 15 articles. Outcome assessor blinding was implemented in 17studies to reduce measurement bias. The risk of bias due to incomplete outcome data was rated as high in 4 studies (attrition rate >15% or imbalanced attrition between groups), and the risk of selective reporting was unclear in 5 studies. Ten studies presented an unclear risk of other biases due to reasons such as small sample size or baseline imbalances. Overall, 10 studies were rated as having a low risk of bias, 7 as having a moderate risk, and 2 as having a high risk of bias, as shown in **Figures 2A, B**.

## 5 Meta-analysis

### 5.1 Analysis of 6-MWT

**Figure 3:**
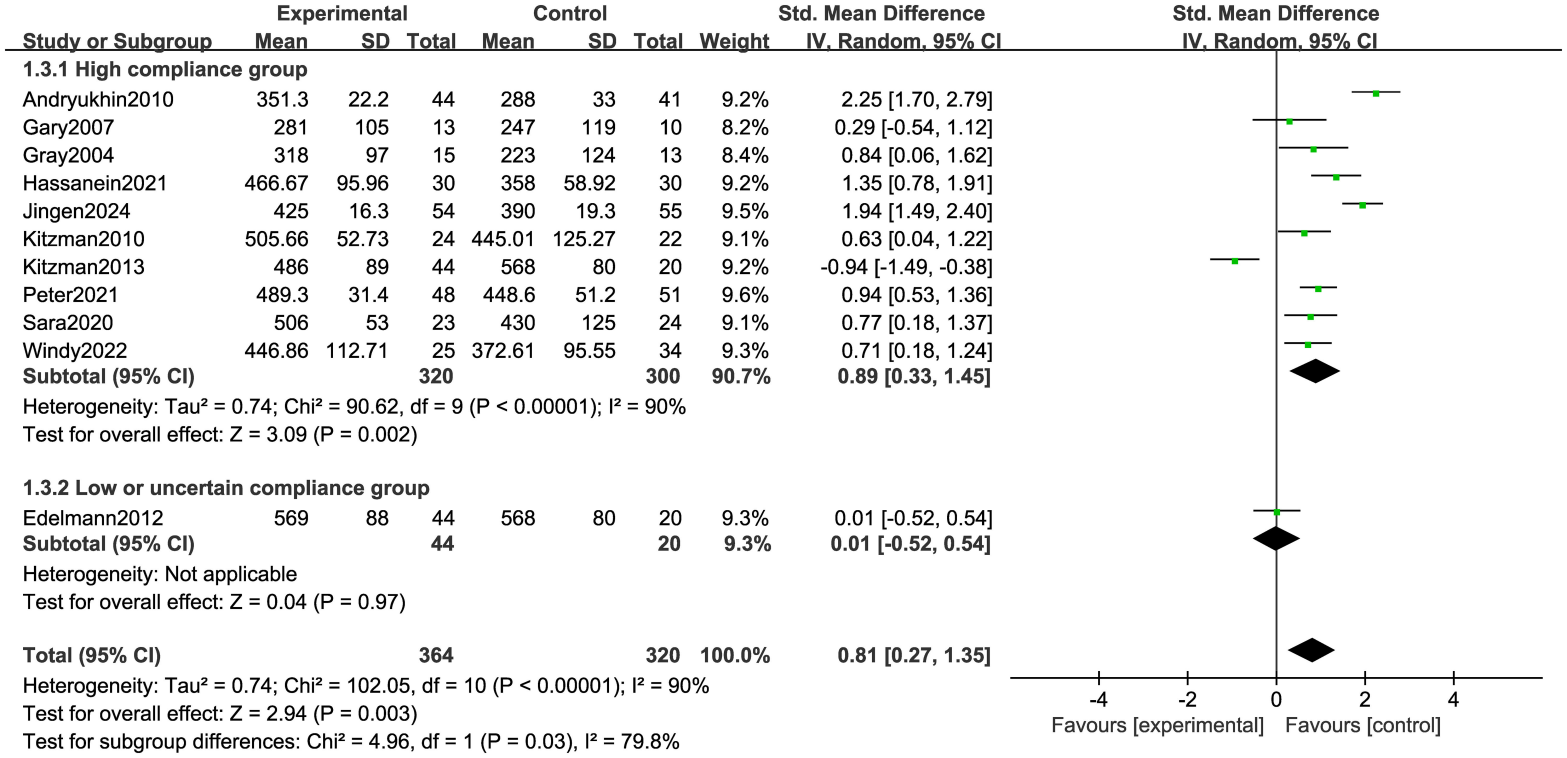
Forest plot of SMD for 6-minute walk test (6-MWT) stratified by intervention compliance (random-effects model). Intervention significantly improved 6-MWT performance only in the high-compliance subgroup; subgroup difference was statistically significant, with high interstudy heterogeneity (I^2^=90%).

This meta-analysis included 11 studies involving 684 patients with HFpEF, all reporting the 6-MWT as the outcome measure. A random-effects model was applied due to substantial between-study heterogeneity (I^2^ = 90%). The overall pooled SMD was 0.81 (95% CI: 0.27–1.35), indicating a beneficial effect of exercise training on 6-MWT performance. The 95% prediction interval calculated using Higgins et al.’s method was −1.181 to 2.797 (see **Supplementary Material S1**).Subgroup analysis based on adherence to ACSM exercise guidelines showed: High-compliance subgroup: pooled SMD = 0.89 (95% CI: 0.33–1.45);Low/uncertain-compliance subgroup: pooled SMD = 0.01 (95% CI: −0.52–0.54).The difference between the two subgroups was statistically significant (P = 0.03, I^2^ = 79.8%) (**Figure 3**).These findings suggest that exercise training improves functional capacity in patients with HFpEF, and high adherence to standardized ACSM exercise regimens yields greater clinical benefits.

### 5.2 Analysis of peak VO_2_

**Figure 4:**
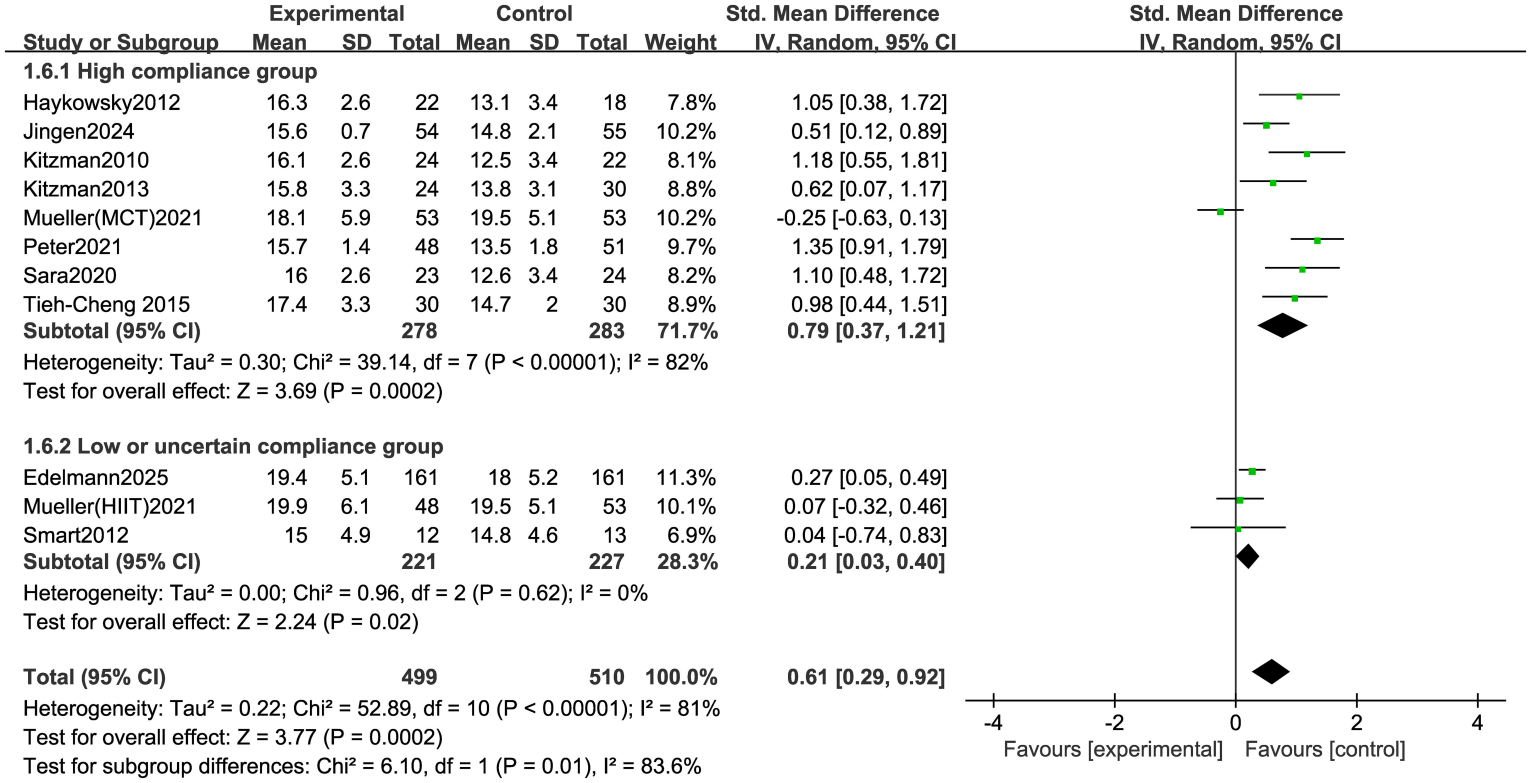
Forest plot of SMD for peak oxygen consumption (peak VO_2_) stratified by intervention compliance (random-effects model). Intervention significantly elevated peak VO_2_ in both subgroups; efficacy was stronger in high-compliance group, with significant subgroup difference (P=0.01). Overall high interstudy heterogeneity (I^2^=81%).

This meta-analysis included 11 studies, involving a total of 1009 patients with HFpEF, with peak VO_2_ as the outcome measure. Due to significant heterogeneity among the studies (I^2^ = 81%), and the analysis was conducted using a random effects model. The overall pooled SMD was 0.61 (95% CI: 0.29 - 0.92), suggesting that exercise training has a positive effect on improving peak VO_2_. The 95% prediction interval calculated using Higgins et al.’s method was −0.547 to 1.762 (see **Supplementary Material S2**). Based on the ACSM exercise adherence, a subgroup analysis was conducted. The results showed that for the high adherence subgroup, the SMD was 0.79, (95% CI: 0.37 - 1.21, I^2^ = 82%), the low/uncertain adherence sub-group combined with an SMD of 0.21 (95% CI: 0.03 - 0.40, I^2^ = 0%), indicating that exercise benefits were observed in both levels of compliance. However, the effect size was significantly larger in the high-compliance subgroup, and the difference between the two groups was statistically significant (P = 0.01, I^2^ = 83.6%) (**Figure 4**).The above results indicate that exercise training can increase the peak VO_2_ of patients with HFpEF, and the benefits are more significant in patients who strictly follow the ACSM standard exercise protocol.

### 5.3 Analysis of total score of MLHFQ

**Figure 5:**
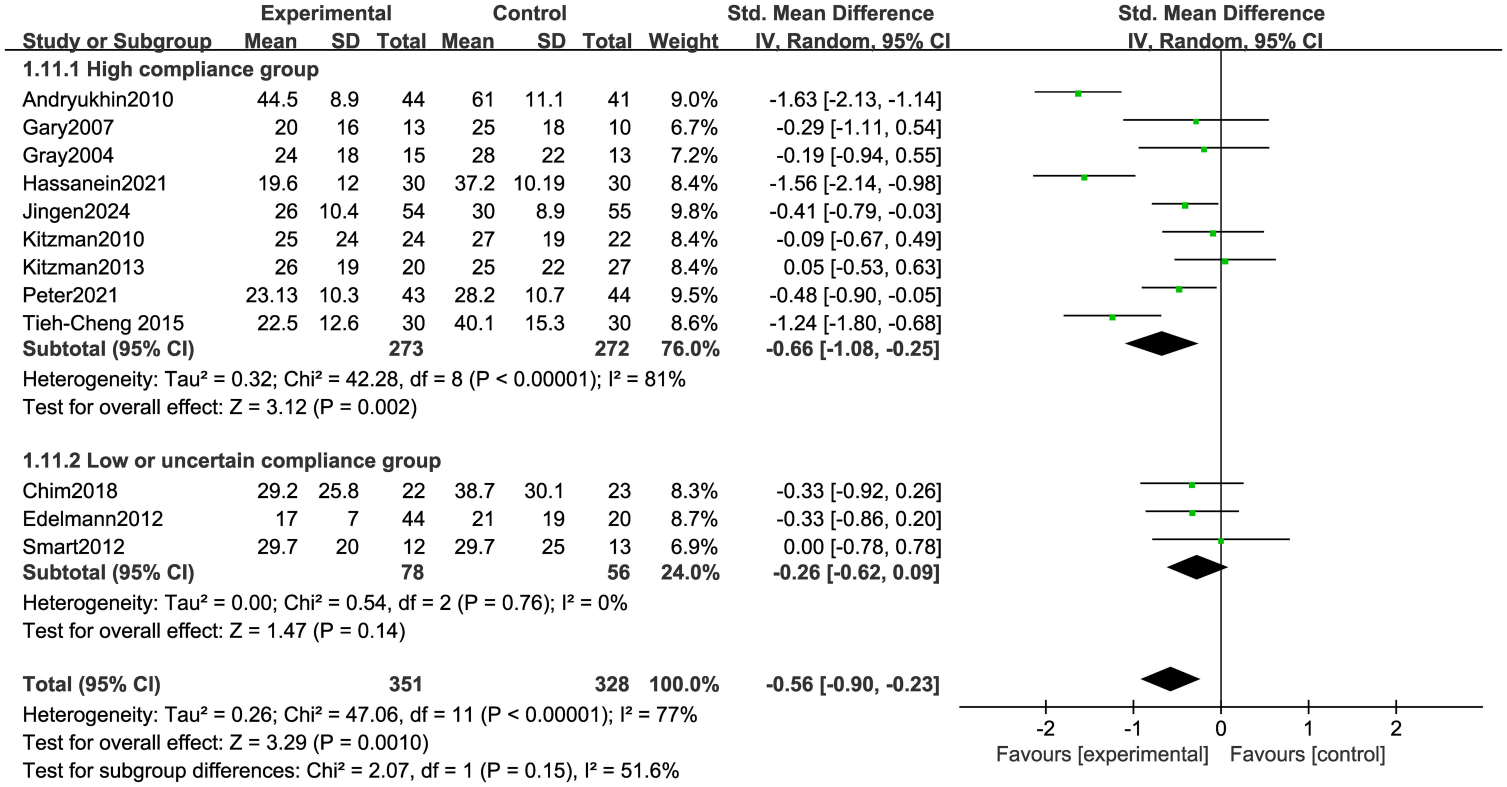
Forest plot of SMD for the total score of the Minnesota Living with Heart Failure Questionnaire (MLHFQ total score) stratified by intervention compliance (random-effects model). Intervention significantly reduced MLHFQ scores only in the high-compliance subgroup; no significant subgroup difference was observed, with overall moderate-to-high interstudy heterogeneity (I^2^=77%).

This meta-analysis included 12 studies involving 679 patients with HFpEF, all reporting MLHFQ score as the outcome measure. A random-effects model was applied due to substantial between-study heterogeneity (I^2^ = 77%). The overall pooled SMD was -0.56 (95% CI: -0.90 to -0.23), indicating a beneficial effect of exercise training on MLHFQ score. The 95% prediction interval calculated using Higgins et al.’s method was -1.786 to 0.664 (see **Supplementary Material S3**).Subgroup analysis based on ACSM exercise adherence showed: High-compliance subgroup: pooled SMD = -0.66 (95% CI: -1.08 to -0.25, I^2^ = 81%); Low/uncertain-compliance subgroup: pooled SMD = -0.26 (95% CI: -0.62 to 0.09, I^2^ = 0%). The difference between the two subgroups was not statistically significant (P = 0.15, I^2^ = 51.6%) (**Figure 5**).These findings suggest that exercise training improves quality of life in patients with HFpEF, with a larger magnitude of improvement observed in those with high adherence to standardized ACSM exercise regimens.

### 5.4 Analysis of LVEF

**Figure 6:**
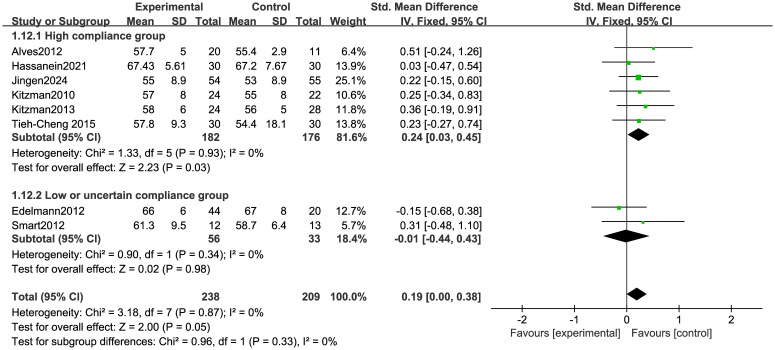
Forest plot of SMD for left ventricular ejection fraction (LVEF) stratified by intervention compliance (fixed-effects model). Intervention showed significant positive effect only in the high-compliance subgroup; no significant inter-subgroup difference was found, and no interstudy heterogeneity existed (I^2^=0%).

This meta-analysis included 7 studies involving 447 patients with HFpEF, all reporting LVEF as the outcome measure. A fixed-effects model was applied due to low between-study heterogeneity (I^2^ = 0%). The overall pooled SMD was 0.19 (95% CI: 0.00 to 0.38), indicating a small but statistically significant improvement in LVEF with exercise training. The 95% prediction interval calculated using Higgins et al.’s method was -0.039 to 0.426 (see **Supplementary Material S4**). Subgroup analysis based on ACSM exercise adherence showed: High-compliance subgroup: pooled SMD = 0.24 (95% CI: 0.03 to 0.45, I^2^ = 0%); Low/uncertain-compliance subgroup: pooled SMD = -0.01 (95% CI: -0.44 to 0.43, I^2^ = 0%). The difference between the two subgroups was not statistically significant (P = 0.33, I^2^ = 0%) (**Figure 6**).These findings suggest that while exercise training may produce a small improvement in LVEF in patients with HFpEF, this effect appears to be limited to those with high adherence to standardized ACSM exercise regimens.

### 5.5 Analysis of E/e’ radio

**Figure 7:**
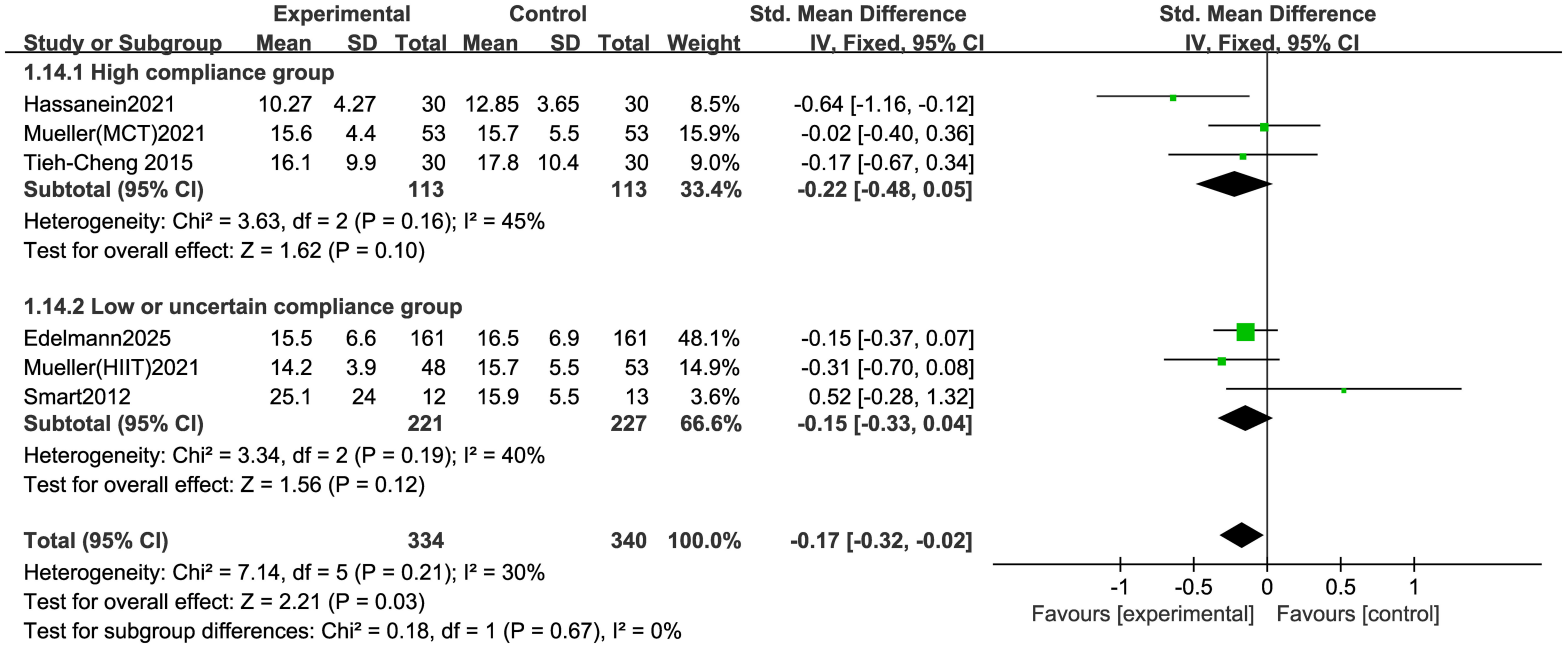
Forest plot of SMD for the ratio of mitral early diastolic inflow velocity to mitral annular early diastolic velocity(E/e’ radio) stratified by intervention compliance (fixed-effects model). Neither subgroup exhibited significant intergroup difference; overall pooled analysis indicated a mild significant benefit of intervention, with no statistical discrepancy between subgroups and low interstudy heterogeneity (I^2^=30%).

This meta-analysis included 6 studies involving a total of 674 patients with HFpEF. All studies used the ratio of E/e’ radio as the outcome measure. Due to the low heterogeneity among the studies (I² = 30%), a fixed-effect model was used. The overall pooled SMD was -0.17 (95% CI: -0.32 to -0.02), suggesting that exercise training could slightly reduce the E/e’ radio level. The 95% prediction interval calculated using Higgins et al.’s method was -0.384 to 0.043 (see **Supplementary Material S5**). The subgroup analysis based on the exercise adherence classification of the ACSM showed: High adherence subgroup: pooled SMD = -0.22 (95% CI: -0.48 to -0.33, I² = 45%); Low/uncertain adherence subgroup: pooled SMD = -0.22 (95% CI: -0.15 to -0.33, I² = 40%). There was no statistically significant difference between the two groups (P = 0.67, I^2^ = 0%) (**Figure 7**). The above results indicate that exercise training can slightly improve the E/e’ radio level in patients with HFpEF, but the improvement is limited, and the benefits do not show significant differences due to variations in exercise adherence.

### 5.6 Analysis of E/A radio

**Figure 8:**
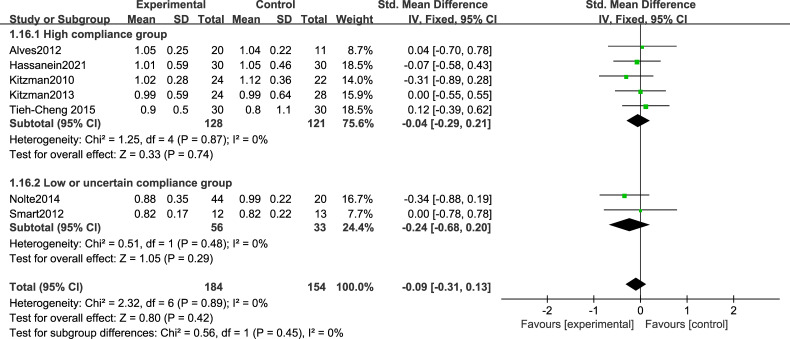
Forest plot of SMD for early diastolic transmitral flow velocity / Atrial systolic transmitral flow velocity ratio(E/A radio) stratified by intervention compliance (fixed-effects model). No significant intergroup difference was observed in either subgroup or overall pooled analysis; no statistical discrepancy existed between subgroups, and no interstudy heterogeneity existed (I^2^=0%).

This meta-analysis included 7 randomized controlled trials involving 338 patients with HFpEF. The primary outcome measure was the E/A ratio. Given the low between-study heterogeneity (I^2^ = 0%), a fixed-effects model was adopted for pooled analysis. The overall SMD was -0.09 (95% CI: -0.31 to 0.13), indicating no significant effect of exercise training on the E/A ratio. The 95% prediction interval calculated using Higgins et al.’s method was -0.370 to 0.192 (see **Supplementary Material S6**). Subgroup analysis stratified by adherence to ACSM exercise guidelines yielded the following results: High-compliance subgroup: pooled SMD = -0.04 (95% CI: -0.29 to 0.21, I^2^ = 0%); Low/uncertain-compliance subgroup: pooled SMD = -0.24 (95% CI: -0.68 to 0.20, I^2^ = 0%). No statistically significant difference was observed between the two subgroups (P = 0.45, I^2^ = 0%). (**Figure 8**). Collectively, these findings suggest that exercise training does not appear to induce a meaningful change in the E/A ratio among patients with HFpEF, regardless of adherence to standardized ACSM exercise regimens.

### 5.7 Analysis of VE/VCO_2_ slope

**Figure 9:**
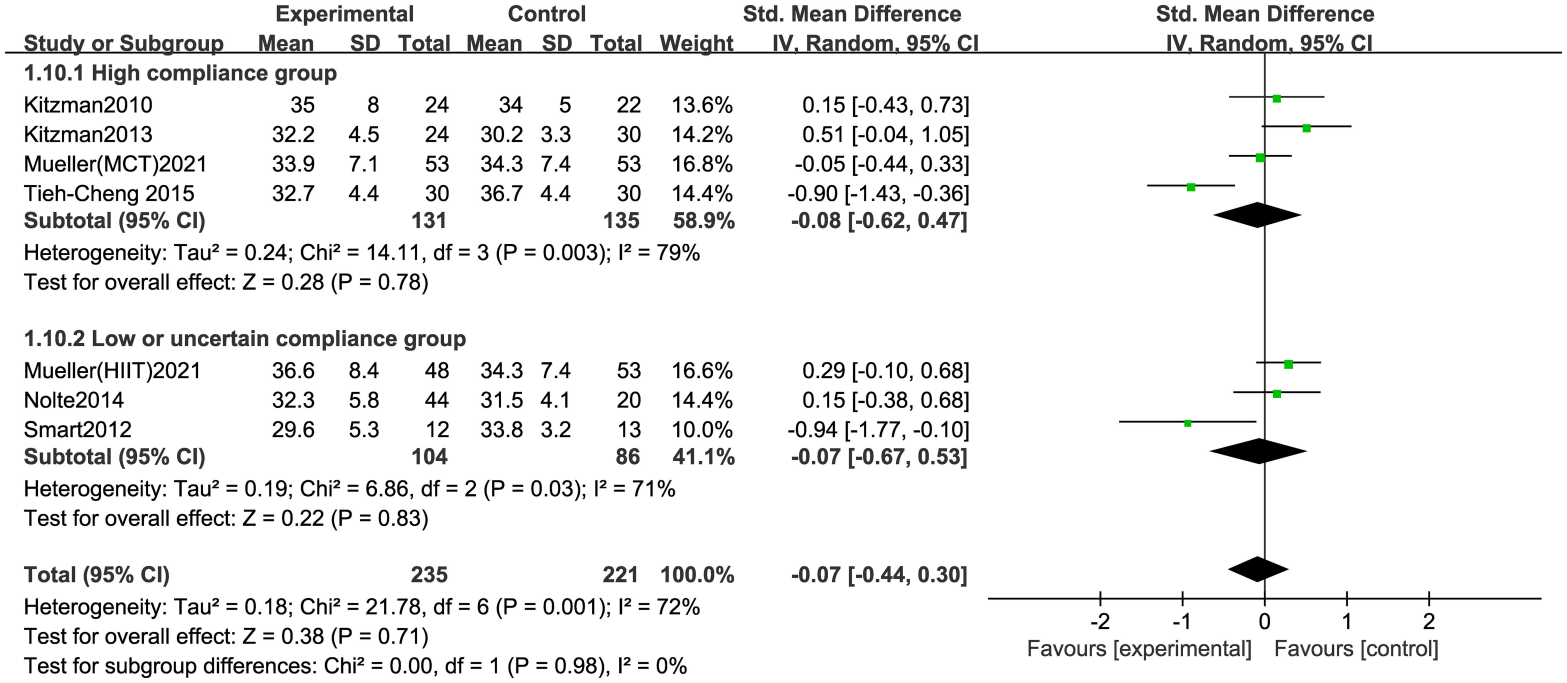
Forest plot of SMD for ventilation / Carbon Dioxide production slope(VE/VCO2 slope) stratified by intervention compliance (random-effects model). No significant intergroup difference was found in two subgroups and overall pooled analysis; no difference existed between subgroups, with moderate-to-high interstudy heterogeneity (I^2^=72%).

This meta-analysis included 7 randomized controlled trials involving 456 patients with HFpEF, all reporting the VE/VCO_2_ slope as the outcome measure. A random-effects model was applied due to substantial between-study heterogeneity (I^2^ = 72%). The overall pooled SMD was -0.07 (95% CI: -0.44 to 0.30), indicating no significant effect of exercise training on the VE/VCO_2_ slope. The 95% prediction interval calculated using Higgins et al.’s method was -1.371 to 1.215 (see **Supplementary Material S7**).Subgroup analysis stratified by adherence to ACSM exercise guidelines yielded the following results: High-compliance subgroup: pooled SMD = -0.08 (95% CI: -0.62 to 0.47, I^2^ = 79%); Low/uncertain-compliance subgroup: pooled SMD = -0.07 (95% CI: -0.67 to 0.53, I^2^ = 71%). No statistically significant difference was observed between the two subgroups (P = 0.98, I^2^ = 0%)(**Figure 9**). Collectively, these findings suggest that exercise training does not appear to induce a meaningful change in the VE/VCO_2_ slope among patients with HFpEF, regardless of adherence to standardized ACSM exercise regimens.

### 5.8 Analysis of deceleration time

**Figure 10:**
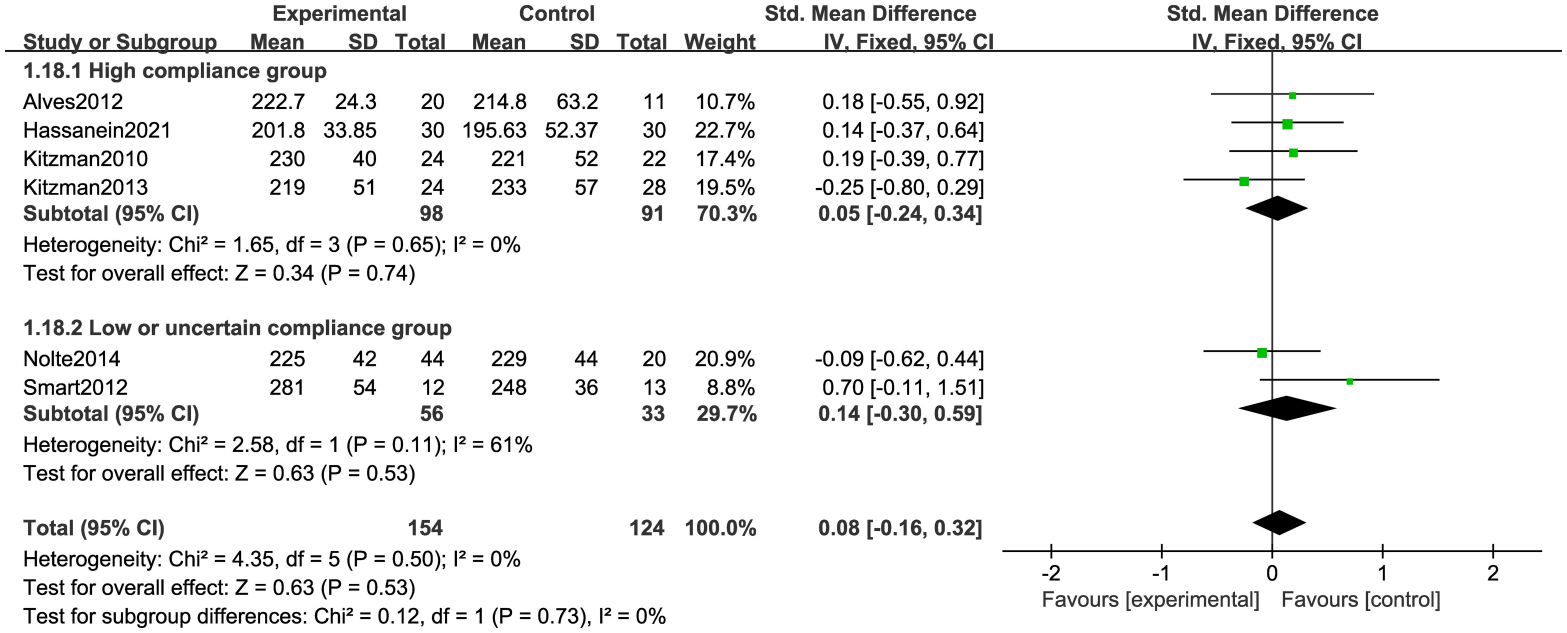
Forest plot of SMD for deceleration time(DT) stratified by intervention compliance (fixed-effects model). No significant intergroup difference was detected in both subgroups and overall pooled analysis; subgroup difference was non-significant, and overall interstudy heterogeneity was zero (I^2^=0%).

This meta-analysis included 6 RCT involving 278 patients with HFpEF, all reporting DT as the outcome measure. Due to the low heterogeneity among the studies (I^2^ = 0%), a fixed-effect model was used. The overall pooled SMD was 0.08 (95% CI: -0.16 to 0.32), indicating no significant effect of exercise training on the DT. The 95% prediction interval calculated using Higgins et al.’s method was -0.256 to 0.417 (see **Supplementary Material S8**). Subgroup analysis stratified by adherence to ACSM exercise guidelines yielded the following results: High-compliance subgroup: pooled SMD = 0.05 (95% CI: -0.24 to 0.34, I^2^ = 0%); Low/uncertain-compliance subgroup: pooled SMD = 0.14 (95% CI: -0.30 to 0.59, I^2^ = 61%). No statistically significant difference was observed between the two subgroups (P = 0.73 I^2^ = 0%) (**Figure 10**). Collectively, these findings suggest that exercise training does not appear to induce a meaningful change in the DT among patients with HFpEF, regardless of adherence to standardized ACSM exercise regimens.

## 6 Publication bias, sensitivity analysis and GRADE assessment

**Figure 11:**
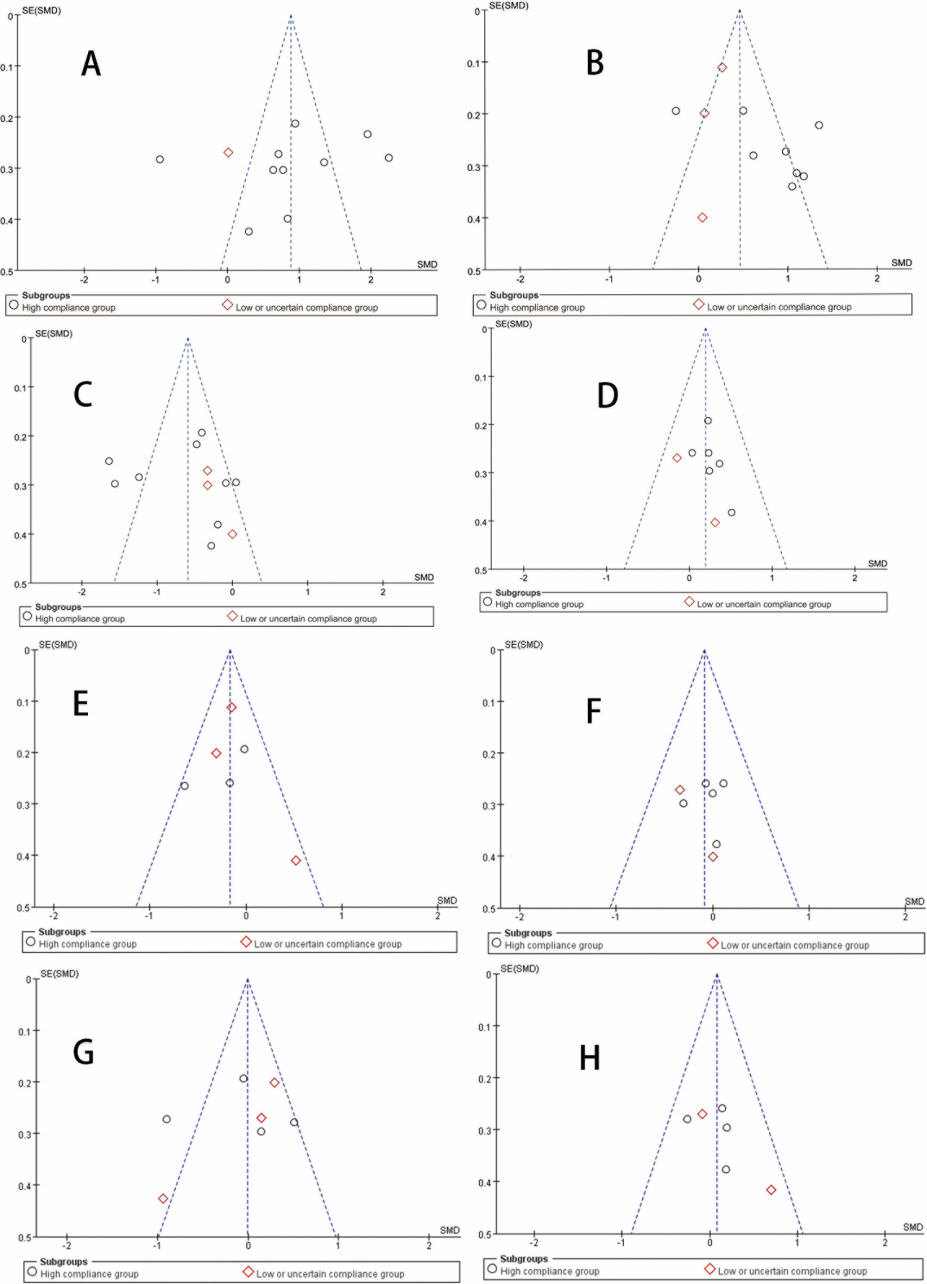
Funnel plots assessing publication bias across all outcomes. The plots exhibited approximate symmetry, suggesting no substantial publication bias. **(A)** 6-MWT; **(B)** peak VO_2_; **(C)** total MLHFQ score; **(D)** LVEF; **(E)** E/e′ ratio; **(F)** E/A ratio; **(G)** VE/VCO_2_ slope; **(H)** deceleration time. SMD, standardized mean difference; SE, standard error.

We evaluated the publication bias through funnel plots (**Figure 11**). These plots show approximate symmetry, indicating no significant publication bias. Through sensitivity analysis by eliminating one by one, we found that no single literature had a significant impact on the overall structure, suggesting that the overall structure is stable. We further applied the GRADE (Grading of Recommendations Assessment, Development and Evaluation) framework to assess the certainty of evidence for all outcomes. Since all included studies were RCTs, the initial evidence certainty was set as High. Five key domains were assessed: risk of bias, inconsistency, indirectness, imprecision, and publication bias. Collectively, the included RCTs did not have serious methodological bias. Blinding of participants and researchers was not fully implemented due to the inherent characteristics of exercise interventions, which was rated as unclear risk; nevertheless, such limitations were not regarded as critical flaws, so no downgrade was applied for the risk of bias domain. Additionally, no indirectness was identified across all analyses. The GRADE scores for each outcome are shown in **Table 5**.

**Table 5:** GRADE Quality assessment of outcomes.

Outcome indicator	Pooled effect size (SMD, 95% CI)	P value	Heterogeneity (I2)	Initial GRADE grade	Reasons for downgrade	Final GRADE grade
6-MWT	0.81(0.27, 1.35)	0.003	90%	High	Severe between-study heterogeneity	Moderate
Peak VO2	0.61 (0.29, 0.92)	0.0002	81%	High	Severe between-study heterogeneity	Moderate
MLHFQ score	−0.56 (−0.90, −0.23)	0.001	77%	High	Severe between-study heterogeneity	Moderate
LVEF	0.19 (0.00, 0.38)	0.05	0%	High	Marginal statistical significance with insufficient precision	Moderate
E/e’ ratio	−0.17 (−0.32, 0.02)	0.03	30%	High	Marginal statistical significance with insufficient precision	Moderate
E/A ratio	−0.09 (−0.31, 0.13)	>0.05	0%	High	No statistical significance	Moderate
VE/VCO2 slope	−0.07 (−0.44, 0.30)	>0.05	72%	High	① Severe between-study heterogeneity ② No statistical significance	Low
DT	0.08 (−0.16, 0.32)	>0.05	0%	High	No statistical significance	Moderate

GRADE certainty: High, further research is very unlikely to change our confidence; Moderate, further research may change our confidence; Low, further research is very likely to change our confidence. For the 6-MWT, Peak VO_2_, and total score of the MLHFQ, substantial between-study heterogeneity led to a one-level downgrade, with the final evidence certainty rated as Moderate. For LVEF, E/e’ ratio, E/A ratio and deceleration time (DT), heterogeneity was low to mild. These outcomes showed marginal or non-significant statistical results (classified as imprecision), one-level downgrade was conducted, and the final certainty was also Moderate. Differently, the VE/VCO_2_ slope was downgraded by two levels on account of both substantial heterogeneity and non-significant pooled results, and its final evidence certainty was rated as Low.

## 7 Discussion

This meta-analysis reviewed19 RCTs involving 1490 participants, including 754 in the experimental group and 736 in the control group. This study is stratify based on the ACSM exercise prescription compliance and explore the impact of following the ACSM exercise intervention on exercise capacity, quality of life, and left ventricular function in patients with HFpEF. The results showed that the exercise intervention following the ACSM protocol could significantly improve the exercise capacity (Peak VO_2_, 6-MWT) and quality of life (total score of MLHFQ) of patients, and showed a marginally significant positive trend in LVEF and diastolic function indicators (E/e’ ratio). Notably, the intervention with high ACSM compliance (compliance ≥ 70%) had more significant benefits in improving exercise capacity and quality of life. This study indicates that high-compliance implementation of the ACSM exercise prescription is a safe and effective strategy for improving the functional status of HFpEF patients, providing high-level evidence for the clinical development of individualized exercise rehabilitation programs.

The core clinical characteristics of HFpEF are reduced exercise tolerance and impaired quality of life, manifested as dyspnea, decreased Peak VO_2_, and diminished quality of life (Haykowsky et al., 2012; Salzano et al., 2021). The conclusions of this study align with the majority of existing evidence, indicating that exercise training provides clear benefits for HFpEF patients. Several meta-analyses have confirmed that exercise can increase patients’ peak oxygen consumption and 6-minute walk distance (Leggio et al., 2020; Boulmpou et al., 2022; Cavero-Redondo et al., 2023; Lin et al., 2023), and improve quality of life scores (Pandey et al., 2015; Fukuta et al., 2019; Gomes-Neto et al., 2019; Baral et al., 2024). Regarding cardiac function, some studies suggest that exercise may improve diastolic function indicators such as E/e’ (Pearson et al., 2017; Cavero-Redondo et al., 2023; Siddiqi et al., 2023), which is consistent with the positive trend observed in our study. However, existing evidence also exhibits some heterogeneity. For example, some studies have not found a significant improvement in the E/e’ ratio (Fukuta et al., 2019) or ventilatory efficiency (VE/VCO_2_ slope) (Gomes-Neto et al., 2019; Siddiqi et al., 2023) with exercise, and some have even reported no significant effect on either left ventricular systolic or diastolic function indicators (Pandey et al., 2015). These discrepancies may stem from variations in the type, intensity, and dosage of exercise prescriptions.

The selection of the optimal exercise plan has become a research hotspot in this field. In recent years, numerous meta-analyses have focused on the effects of exercise intensity and exercise type on patients with HFpEF. For instance, Edwards et al. published a Meta-analysis that specifically compared three modes: moderate-intensity aerobic exercise (MIT), high-intensity interval training (HIIT), and combined aerobic and resistance training (CT). The study found that HIIT significantly improved the left ventricular ejection fraction of HFpEF patients, but did not outperform moderate-intensity continuous training in improving Peak VO2, and combining aerobic exercise with resistance training did not diminish the benefits of aerobic training (Edwards et al., 2023).Gomes-Neto et al. found that high-intensity interval training (HIIT) and the exercise method combining aerobic and resistance training might have more advantages in enhancing exercise capacity (Gomes-Neto et al., 2024). Baral et al. suggested that high-intensity interval training (HIIT) is superior to moderate-intensity continuous training (Baral et al., 2024), while other scholars reached the opposite conclusion (Guo et al., 2022). Therefore, the optimization of various parameters of the exercise plan still requires further research.

Notably, most trials included in the present work adopted standalone aerobic exercise rather than the full multi-component regimens recommended by the American College of Sports Medicine (ACSM). This is a prevalent real-world issue in contemporary clinical practice and relevant trials. Due to limitations in patients’ exercise tolerance, safety concerns and practical operational constraints, most clinical centres and research teams are only able to implement aerobic training alone in routine HFpEF rehabilitation. Nevertheless, aerobic exercise is the core component of ACSM exercise prescriptions, and all single aerobic protocols analyzed in this work fully conformed to ACSM’s specifications for exercise intensity, frequency and duration. Accordingly, this study focused on interventions adhering to the core principles of ACSM, rather than exclusively enrolling trials with complete multi-component training.

The greatest advantage of ACSM-based exercise regimens lies in their scientificity and standardization compared with arbitrary exercise protocols used in early clinical practice. Different from most previous studies that simply verified the general efficacy of exercise, the core novelty of the present study is to answer a more clinically practical question: how to implement exercise programs to achieve better outcomes. As a global authoritative organization in sports medicine, ACSM has formulated detailed exercise prescriptions for patients with cardiovascular diseases, which clearly define the frequency, intensity, duration and types of aerobic, resistance and flexibility training based on patients’ physiological characteristics (Garber et al., 2011). ACSM-standardized protocols strike a good balance between efficacy and safety: they avoid poor therapeutic effects caused by insufficient exercise intensity, and meanwhile reduce the risk of adverse cardiovascular events induced by excessive load. For instance, the moderate-intensity aerobic exercise recommended by ACSM can effectively improve myocardial systolic and diastolic function without overloading the heart (Sesso et al., 2000; Lee et al., 2001; Manson et al., 2002; Tanasescu et al., 2002). Resistance training can strengthen skeletal muscle and enhance exercise endurance, thus producing synergistic effects with aerobic exercise (Upadhya et al., 2015).

The subgroup analysis results of this study demonstrated more prominent benefits brought by medium to high ACSM compliance, highlighting the significance of standardized implementation of exercise prescriptions in the exercise rehabilitation of HFpEF patients. High ACSM compliance (≥70%) was a guarantee for achieving significant improvements in exercise capacity and quality of life, while the benefits in the low compliance group were very limited. This suggests that insufficient or non-standardized exercise intervention (such as inadequate frequency or inappropriate intensity) is difficult to produce definite clinical effects. Although the high compliance group had better improvement values in quality of life and left ventricular ejection fraction (LVEF), the differences compared with the low compliance group did not reach statistical significance, which might be related to the heterogeneity in the intervention period, assessment tools (such as only using the MLHFQ scale for quality of life), and other aspects of the included studies. Notably, even under high compliance conditions, the improvement in the E/e’ ratio by exercise was not significant, suggesting that relying solely on exercise training may have limitations in reversing the core diastolic dysfunction of HFpEF. Future research should explore comprehensive management strategies combining exercise with other targeted treatments.

This study also has certain limitations that should be taken into account when interpreting the results. First, our meta-analysis set left ventricular ejection fraction (LVEF) ≥ 45% as the inclusion criterion for patients with heart failure with preserved ejection fraction (HFpEF), which differs from the LVEF ≥ 50% threshold recommended by current international guidelines. The classification of heart failure based on LVEF has evolved alongside advances in clinical research and guideline revisions. In early heart failure research, the academic community generally defined LVEF < 40% as heart failure with reduced ejection fraction (HFrEF) and LVEF ≥ 40% as HFpEF. This dichotomous standard was first adopted in the classic CHARM trials without an intermediate category (Yusuf et al., 2003). Later landmark studies including TOPCAT, PARAGON-HF, and I-PRESERVE all used LVEF ≥ 45% to enroll HFpEF patients (Carson et al., 2005; Anand et al., 2017; Solomon et al., 2017). Current guidelines clearly recognize a transitional range between heart failure with mildly reduced ejection fraction (HFmrEF) and HFpEF (Meunier-McVey, 2021). To date, the LVEF ≥ 45% cut-off has been widely adopted in numerous published meta-analyses focusing on HFpEF. Strictly limiting inclusion to LVEF ≥ 50% would exclude many high-quality eligible primary studies, reducing the total sample size, lowering statistical power and potentially causing selection bias. We therefore adopted LVEF ≥ 45% uniformly to ensure the comprehensiveness of this systematic review and meta-analysis. However, this approach may create discrepancies between our study population and HFpEF patients defined by current guidelines, so our findings cannot be fully generalized to patients diagnosed according to contemporary clinical criteria. Second, diverse exercise modalities (e.g., Baduanjin, chair exercises) and intervention durations (12 to 72 weeks) across included studies introduced clinical heterogeneity. Third, our assessment of adherence to ACSM guidelines relied solely on objective parameters extracted from study protocols, without incorporating patients’ real-world exercise data, which may lead to assessment bias. Fourth, several included studies had small sample sizes and a high risk of bias, although sensitivity analysis confirmed that our overall results were robust. Fifth, we did not conduct additional subgroup analyses based on exercise patterns or patient baseline characteristics such as age and comorbidities, which limits the precision of individualized exercise guidance. Sixth, the study population may exhibit heterogeneity. A small fraction of patients may have regained normal LVEF following treatment for prior HFrEF. Their underlying mechanisms differ from conventional HFpEF caused by hypertension and high filling pressures, possibly leading to inconsistent responses to exercise. This factor needs to be considered in clinical application. Finally, GRADE assessment revealed that incomplete blinding and substantial between-study heterogeneity reduced the reliability and generalizability of our evidence. More well-designed RCTs are needed to further strengthen the current evidence base.

Despite the above-mentioned limitations, the conclusions of this study still hold significant clinical practice and evidence-based medical implications. The exercise program recommended by ACSM is characterized by low cost, high safety, and easy promotion. When clinical physicians formulate exercise prescriptions for HFpEF patients, they should make individualized adjustments based on the ACSM principles, taking into account the patients’ basic cardiopulmonary function, physical condition, and comorbidities: for patients with better functional status, moderate-intensity aerobic combined with resistance training can be adopted; for elderly, frail or severely ill patients, it is necessary to start from low intensity and short duration, gradually increase, and improve long-term compliance through enhanced supervision, education and follow-up, thereby promoting the transformation of HFpEF management to an integrated model of “medication - exercise rehabilitation”. Based on this study, future research can be further explored in the following directions: (1) conducting large-scale, long-term follow-up randomized controlled trials to verify the impact of standardized ACSM programs on hard endpoint events; (2) developing comprehensive exercise compliance assessment tools that integrate subjective and objective indicators; (3) conducting subgroup analyses at the patient level to identify specific population characteristics that respond best to different exercise programs; (4) comparing the efficacy differences of different exercise combinations (such as pure aerobic vs. combined training) and exploring the synergistic effects of exercise and new drugs (such as SGLT2 inhibitors); (5) conducting pragmatic studies to evaluate the feasibility and effectiveness of this program in real-world clinical settings.

## 8 Conclusion

The exercise guidance provided by ACSM can effectively improve the physical ability and quality of life of patients with HFpEF, as well as some left ventricular function indicators. Moreover, the higher the compliance, the better the functional outcome. In the future, more studies focusing on multi-component exercise and combined treatment are needed to supplement the existing evidence.
