# Supplementary material for: Effects of exercises based on ACSM recommendations on patients with heart failure with preserved ejection fraction: a systematic review and meta-analysis of randomized controlled trials
Source: Front Physiol. 2026 Jul 9;17:1838821. doi: 10.3389/fphys.2026.1838821 (PMC13391323; doi:10.3389/fphys.2026.1838821)
Supplement: Supplementary file 1 [file Supplementaryfile1.zip › Table 1 Search strategy.docx]

| Search | Query |
| --- | --- |
| #1 | "Heart Failure, Diastolic"[Mesh] |
| #2 | "heart failure diastolic"[Title/Abstract] OR "diastolic heart failures"[Title/Abstract] OR "diastolic heart failure"[Title/Abstract] OR "heart failure preserved ejection fraction"[Title/Abstract] OR "heart failure normal ejection fraction"[Title/Abstract] |
| #3 | #1 OR #2 |
| #4 | "Exercise"[MeSH Terms] |
| #5 | "Exercise"[Title/Abstract] OR "Walking"[Title/Abstract] OR "nordic walking"[Title/Abstract] OR "Exercises"[Title/Abstract] OR "physical activity"[Title/Abstract] OR "activities physical"[Title/Abstract] OR "activity physical"[Title/Abstract] OR "physical activities"[Title/Abstract] OR "exercise physical"[Title/Abstract] OR "exercises physical"[Title/Abstract] OR "physical exercise"[Title/Abstract] OR "physical exercises"[Title/Abstract] OR "exercise aerobic"[Title/Abstract] OR "aerobic exercise"[Title/Abstract] OR "aerobic exercises"[Title/Abstract] OR "exercises aerobic"[Title/Abstract] OR "exercise training"[Title/Abstract] OR "exercise trainings"[Title/Abstract] OR "training exercise"[Title/Abstract] OR (("education"[MeSH Subheading] OR "education"[All Fields] OR "Training"[All Fields] OR "education"[MeSH Terms] OR "train"[All Fields] OR "train s"[All Fields] OR "trained"[All Fields] OR "training s"[All Fields] OR "Trainings"[All Fields] OR "trains"[All Fields]) AND "Exercise"[Title/Abstract]) OR "training resistance"[Title/Abstract] OR "strength training"[Title/Abstract] OR "training strength"[Title/Abstract] OR "Balance"[Title/Abstract] OR "Ambulation"[Title/Abstract] OR "stair climbing"[Title/Abstract] OR "walking nordic"[Title/Abstract] OR "pole walking"[Title/Abstract] OR "walking pole"[Title/Abstract] |
| #6 | #5 OR #4 |
| #7 | #6 AND #3 |

Table 1 Search strategy on PubMed
