# Supplementary material for: Effects of exercises based on ACSM recommendations on patients with heart failure with preserved ejection fraction: a systematic review and meta-analysis of randomized controlled trials
Source: Front Physiol. 2026 Jul 9;17:1838821. doi: 10.3389/fphys.2026.1838821 (PMC13391323; doi:10.3389/fphys.2026.1838821)
Supplement: Supplementary file 1 [file Supplementaryfile1.zip › Supplementary material S1-S8.docx]

**Supplementary material S1.** 95% prediction interval (−1.181, 2.797) of pooled effect for 6-MWT produced by Stata 18.0. The wide interval indicated large cross-study variation, and the true effect of exercise could be negative or markedly positive in future trials.





**Supplementary material S2.** 95% prediction interval (−0.547, 1.762) of pooled effect for peak VO₂ produced by Stata 18.0. The wide interval indicated large cross-study variation, and the true effect of exercise could range from slightly negative to strongly positive in future trials.





**Supplementary material S3.** 95% prediction interval (−1.786, 0.664) of pooled effect for MLHFQ total score produced by Stata 18.0. The wide interval indicated large cross-study variation, and the true effect of exercise could range from markedly negative to weakly positive in future trials.





**Supplementary material S4.** 95% prediction interval (−0.039, 0.426) of pooled effect for LVEF produced by Stata 18.0. The narrow interval indicated minimal cross-study variation, and the true effect of exercise would fluctuate slightly from weakly negative to moderately positive in future trials.





**Supplementary material S5.** 95% prediction interval (−0.384, 0.043) of pooled effect for E/e′ ratio produced by Stata 18.0. The narrow interval indicated minimal cross-study variation, and the true effect of exercise would fluctuate slightly from moderately negative to weakly positive in future trials.





**Supplementary material S6.** 95% prediction interval (−0.370, 0.192) of pooled effect for E/A ratio produced by Stata 18.0. The narrow interval indicated minimal cross-study variation, and the true effect of exercise would fluctuate slightly from moderately negative to weakly positive in future trials.





**Supplementary material S7.** 95% prediction interval (−1.371, 1.215) of pooled effect for VE/VCO₂ slope produced by Stata 18.0. The wide interval indicated large cross-study variation, and the true effect of exercise could range from markedly negative to moderately positive in future trials.





**Supplementary material S8.** 95% prediction interval (−0.256, 0.417) of pooled effect for deceleration time produced by Stata 18.0. The narrow interval indicated minimal cross-study variation, and the true effect of exercise would fluctuate slightly from weakly negative to moderately positive in future trials.
